# The American Dental Association

**Published:** 1886-03

**Authors:** 


ARTICLE VI.

### THE AMERICAN DENTAL ASSOCIATION.

### WHERE SHALL THE NEXT MEETING BE HELD, CHICAGO OR SAN FRANCISCO.

At an informal meeting in Buffalo, in December, at which six members of the Executive Committee were present, a vote was taken resulting in five for Chicago, one for

Buffalo. It was decided after the vote to leave the final decision and completion of arrangements with the Committee of Arrangements, they to be governed by circumstances in the final decision. Since more recently, a proposition to hold it in San Francisco has been made, it is deemed best to submit the question to the profession.

The reasons for going to California have already been so fully given in an article in the dental journals, and more recently in a circular letter, that it is unnecessary for me to take space to present them again. They read well and make us all feel like saying Go? *Why, of course!* But facts are hard things to knock against, and it will be found much easier for the majority of the dental profession to say to some other fellow, "Go!" than it will be to muster the money and spare the time to go himself. The rank and file of our profession are not rich men. The question of expense has to be considered by many.

A large per cent. are young men that need the meeting, and the Association needs them. How many will feel that they can spare the time and money necessary for so long a trip? Calculate a week to go, a week to return, the best of a week for the meeting, and two weeks to see California and the Great West, including points of interest on the route, and five weeks are gone. Fare from Chicago, round trip, $62.50. Meals and sleeper about $5.00 per day. Expenses $5.00 per day at lowest estimate at a time when the city and surrounding country are thronged with the Grand Army and thousands of strangers. Add to this, extra railroad and steamboat fare for all side trips to points of interest, and the extras that you can never plan for, and it is easy to see by all who have traveled much that $300 would be a *very* low estimate for the expense, besides at least five weeks of time. Those who have been to California all coincide in the statement that one can not see enough of California and the West to pay them for going without a greater expenditure of time and money than we have named. It is well known that July and August are the most disagreeable months in which to make the trip to California, and we see the country at its worst. These are the months when Californians get away if they can. Since learning the desire of some that the American Dental Association should be held in San Francisco, the California Dental Societies have very courteously invited us, and we are sure their hospitality will be fully appreciated by all, but there are times when we can not afford to accept even hospitality. This is one. The association would lose too much. We could not hope for any large accession of new members, nor for the new ones gained to often met with us from so great a distance, and we should lose many.

Chicago was selected on account of its being central, easily accessible from all parts of the country at low railroad rates, and of its having unsurpassed hotel accommodations, and cool summers. The fact that a hundred of the new members elected last year were Western men and should be held, also entered into the decision. Your Committee have been quietly planning and working since the last meeting to insure at our next the largest attendance and most successful meeting ever yet held, feeling that each meeting should be an advance upon the one that precedes in points of numbers and interest; that we ought not only to hold the new members gained last year, but that we should add as many more at our coming session. It is too soon to complete definite railroad arrangements, but if the decision is for Chicago, we expect to bring them within the reach of all.

At a meeting of the Chicago members of the American Dental Association and of the Chicago Dental Society, called to ascertain the views of the profession here March 17th, the following Resolution was adopted by a vote of twenty-seven to five:

RESOLVED, *That it is the sense and desire of this body that the next meeting of the American Dental Association should be held in Chicago, and that we extend to the Association a most cordial and hearty invitation to meet mith us.*

AN EXCURSION TO CALIFORNIA AFTER THE MEETING.

Your committee are assured by the railroad authorities that they can have equally as favorable rates in all respects for an excursion upon close of the meeting, if any considerable number wish to go to California, as are promised for the Association. The committee will see that *no pains are spared* to make such an excursion a success, if enough signify their wish to go to warrant making the arrangements. Thus, none who wish to go will be deprived of seeing Caliifornia at the reduced rates while great numbers will not be deprived of attending the association because they can not afford a trip to California. It is a serious question whether the society has a right to hold its meetings beyond the reach of so large a class. Let us remember that our association is a scientific body intended to reach and benefit *the great body of the profession* as far as possible.

There has been so much discussion of the whole subject in the Committee and out, and the Committee are at such distances from each other that it is impossible to get a united expression of views *in time for the Journals*, as promised, hence, I submit this as an expression of a part of the Committee. Am very sure that each member of the Committee wishes to do only the very best thing for the Association, and to carry out the wishes of its members, and your votes will show us what those are and greatly facilitate the work of the Committee.

Please answer *promptly* by letter or postal the following questions:

In your judgment, should the meeting be held in Chicago or San Francisco?

Do you expect to attend the meeting if held in San Francisco?

Do you expect to attend the meeting if held in Chicago?

If the meeting is held in Chicago, will you probably go to California after the meeting is over, if an excursion is decided upon?

J. N. CROUSE, *Chairman* Ex. Com. & Cóm. of Arr.

2101 Michigan Ave., Chicago. Ill.

